# An Analysis of Running Impact on Different Surfaces for Injury Prevention

**DOI:** 10.3390/ijerph20146405

**Published:** 2023-07-20

**Authors:** Amelia Ferro-Sánchez, Adrián Martín-Castellanos, Alfonso de la Rubia, Abraham García-Aliaga, Mónica Hontoria-Galán, Moisés Marquina

**Affiliations:** 1 Sport and Training Research Group, Department of Sports, Faculty of Physical Activity and Sport Sciences, Universidad Politécnica de Madrid (UPM), 28040 Madrid, Spain; 2 Department of Physical Activity and Sports Science, Alfonso X El Sabio University (UAX), 28691 Madrid, Spain

**Keywords:** accelerometry, accelerations, synthetic track, grass, concrete

## Abstract

The impact that occurs on the runner’s foot when it lands on the ground depends on numerous factors: footwear, running technique, foot strike and landing pattern, among others. However, the surface is a decisive factor that can be selected by the runner to improve their sports practice, thereby avoiding injuries. This study aimed to assess the number and magnitude of accelerations in impact (produced by the runner when their foot strikes the ground) on three different surfaces (grass, synthetic track, and concrete) in order to know how to prevent injuries. Thirty amateur runners (age 22.6 ± 2.43 years) participated in the study. They had to run consecutively on three different surfaces at the same speed, with a three axis-accelerometer placed on the sacrum and wearing their own shoes. The results showed that the running impacts differed based on the type of surface. Higher mean acceleration (MA) and mean peak acceleration (PA) in the impacts were observed on concrete compared to the other two surfaces. There were small differences for MA: 1.35 ± 0.1 g (concrete) vs. 1.30 ± 0.1 g (synthetic track) SD: 0.43 (0.33, 0.54) and 1.30 ± 0.1 g (grass) SD: 0.36 (0.25, 0.46), and small differences for PA: 3.90 ± 0.55 g (concrete) vs. 3.68 ± 0.45 g (synthetic track) SD 0.42 (0.21, 0.64) and 3.76 ± 0.48 g (grass) SD 0.27 (0.05, 0.48), implying that greater impacts were produced on concrete compared to synthetic track and grass. The number of peaks of 4 to 5 g of total acceleration was greater for concrete, showing small differences from synthetic track: SD 0.23 (−0.45, 0.9). Additionally, the number of steps was higher on synthetic track (34.90 ± 2.67), and small differences were shown compared with concrete (33.37 ± 2.95) SD 0.30 (−0.25, 0.85) and with grass (35.60 ± 3.94) SD 0.36 (−0.19, 0.91). These results may indicate a change in technique based on the terrain. Given the increasing popularity of running, participants must be trained to withstand the accelerations in impact that occur on different surfaces in order to prevent injuries.

## 1. Introduction

Running is one of the most practiced sports today, and its popularity is increasing, as evidenced by the increase in the number of participants in all the great marathons year after year. This growth in popularity has simultaneously been accompanied by an increase in the incidence of running injuries [1]. Repetitive collisions of the runner’s foot with the ground upon landing could cause a high incidence of chronic overload injuries. Most injuries to runners are associated with the lower limb, with the knee being one of the most common areas (ranging from 7.2% to 50% of injuries). This could be due to the fact that the knee receives the most impact force during running [2]. Injuries of the other parts of the lower limbs, such as the lower leg (shin, calf, Achilles tendon, and heel) had an incidence ranging from 9.0% to 32.2%; the foot ranged from 5.7% to 39.3%, and the upper leg (thigh, hamstring, and quadriceps muscles) ranged from 3.4% to 38.1%. The ankle (from 3.9% to 16.6%) and hip/pelvis (from 3.3% to 11.5%) were the less common sites of lower extremity injuries [2].

Some populations, such as amateur runners, showed an injury prevalence of around 58%, with male runners with and without previous injuries making up 72.4% and 72.6% of this group, respectively [3]. The causes of these injuries have been studied in depth, with the main factors being running shoes, running technique, a high weekly volume of training, or lack of adequate training. Lately, the impact of running surfaces has also been cited as a potential injury risk factor [3,4,5,6]. The impacts on the runner’s foot that occur when the foot lands on the ground depend on numerous factors: footwear, foot strike and landing pattern, running technique, and fatigue, among others. However, the surface is a decisive factor that can be selected by the runner in order to improve their sports practice, thereby avoiding injuries [3,4,5,6]. There is a need for a better understanding of how runners moderate their loading and gait in response to “real-world” surfaces [5].

One of the concepts that should be clarified is the term “impact”; this term encompasses impact force peaks and impact acceleration peaks [7]. The variables related to impact acceleration peaks must be well defined in order to assess the effect of ground reaction forces on the locomotor apparatus of the runners. If runners understand this effect, it will be possible to prevent injuries. Running (and in particular, distance running) is associated with a high number of impacts upon landing; impact force peaks at about 1.5–2.5% of body weight, and impact acceleration peaks at about 8 g. Research has shown that a person who runs 32 km per week can experience up to 1.3 million impacts per year. Although these impacts may not be extreme, the number of impacts could be significant [7]. Shock attenuation and the severity of the impact acceleration are two of the most important variables analyzed in running research because of their potential relationship with injury risk [8,9], fatigue [7,10,11], and running performance [7,11].

One approach to quantify these variables has been the use of instrumented insoles to design cushioned footwear to improve impact attenuation on aggressive surfaces [6,12,13]. Another approach has been the use of accelerometers, used to measure the effect of impact forces received during running [10,14]. These impacts, evaluated through accelerometers, permit the analysis of variables such as impact g-forces (accelerations in the impacts or impact acceleration) and the number of impacts, which can be interpreted in every step.

Accelerometers have been used to assess impact differences between running barefooted and running in shoes [15,16,17], and the modifications needed to adapt footwear [18]. Studies have also analyzed the differences between terrains using accelerometers. Previous research has concluded that the peak acceleration was lower on synthetic track than on concrete [4]. Additionally, lower vertical acceleration peaks were found while running on a softer surface, such as a woodchip trail, when compared to concrete or synthetic track [4,5]. However, in all these studies, the position of the accelerometer has not been identical, and this could affect the data that is registered; this makes it difficult to compare the data. The majority of the studies placed the accelerometers on the tibia; on the proximal anteromedial aspects of the right tibia [10]; on the lateral surface of the distal lower leg [17]; 8 cm above the medial malleolus on the right tibia [4]; and on the tibial tuberosity of the dominant leg [13], among others. A tri-axial accelerometer positioned over the L3 spinous process of the trunk was used to investigate the effects of dynamic stability and dynamic loading during running [5], and a single inertial sensor was situated on the sacrum to identify symmetry in running gait [16]. More studies are needed to analyze accelerations in impact, wherein the accelerometer is placed in the lower part of the spine and closer to the center of mass (CM) (e.g., on the sacrum), where the effect of the impacts would be more attenuated than in the lower limbs; this positioning would also provide more global information on the effect of the impacts on the locomotor system of the runner, and specifically on the spine.

Although impacts vary from surface to surface, amateur runners tend to use the same running shoes over different surfaces [19]. In fact, the main reason that runners change their shoes is the wear and tear of their shoes. This could be considered an influential factor in the development of musculoskeletal injuries due to the runner’s kinematic adaptations to worn-out shoes. However, the effect of different surfaces on the impacts received by an athlete running in the same session at the same speed while wearing the same footwear have not been evaluated, to the best of the author’s knowledge. Such an evaluation could provide valuable information for the selection of the track on which training and/or races should be held, both at amateur and professional levels [20].

Therefore, this study aimed to assess the number and magnitude of the accelerations in impacts produced by runners wearing their own shoes and running consecutively on three different surfaces: grass, synthetic track, and concrete. Another of the features of this study was the innovative location of accelerometers, near to the runner’s center of mass (CM). This could help to select the most suitable surfaces for a workout, which will help trainers and athletes to reduce the risk of injury. The hypothesis for the study is that running on concrete surfaces generates higher accelerations in impacts than running on grass and on a synthetic track, when the runner’s foot strike in landing on the ground.

In this study, we proposed to position the accelerometers close to the CM of the individuals studied in order to record the magnitude and acceleration in the impacts from the most global point of the runner. Considering how these impacts may influence the runner in the race, this may enable trainers to look for facilities to plan training sessions and manage loads in such a way that the risk of injury to athletes can be reduced.

In addition, from an ecological perspective, and taking into consideration what has previously been observed regarding changes of shoes, this research considers that if athletes can carry out this type of field test with the shoes they use in their training sessions, the results will present a more realistic picture of what happens in reality, bringing the results closer to the implementation of actions, which is not as far away as in other studies.

## 2. Materials and Methods

A comparative, descriptive study design was applied wherein the participants had to run on three different surfaces (synthetic track, concrete, and grass) with an accelerometer registering the magnitude of the acceleration in the impacts in each step and on each surface.

### 2.1. Sample

A total of 30 amateur runners, 8 females and 22 males (age 22.6 ± 2.43 years, range 18–24) participated in the study. All of them were Sport Sciences degree students and amateur runners with good technique who were familiar with the facilities in which the study was carried out, and who had at least 2 years of experience. As an exclusion criterion, the athletes had to have no lower limb injuries in the last 2 years. Before the trial, the subjects were informed about the purposes and risks of the investigation, and each participant signed a written informed consent document in accordance with the Declaration of Helsinki [21]. The study was approved by the university’s ethics committee. An a priori sample size was calculated (G*Power software, Version 3.1 [22]) using ANOVA repeated measures within factors (significance level α = 0.05, β = 0.8, and effect size d = 0.25). This resulted in a final minimum sample size of 28 participants.

### 2.2. Methodology

**Figure 1:**
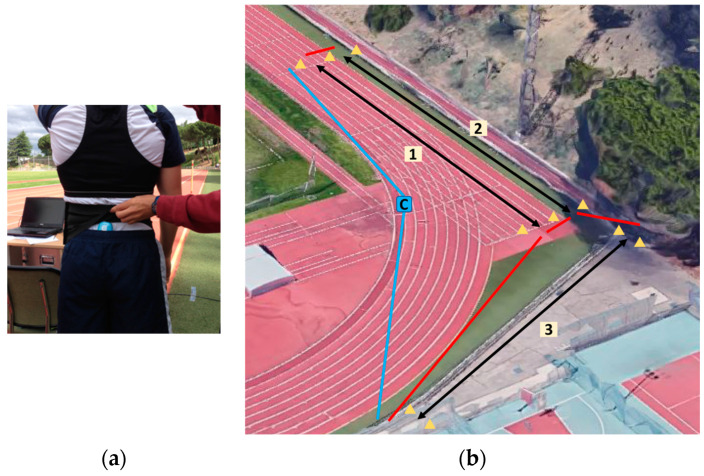
(**a**) The WIMU placed on the runner’s sacrum; (**b**) athletic track: (1) 40 m synthetic track; (2) 40 m grass and (3) 40 m concrete; red lines: transition paths; blue lines: bounded area for the test; C: the control point.

The participants carried a multi-sensor wireless inertial measurement unit (WIMU v. 1.6, Real Track^®^, Almería, Spain), with two three-axis-accelerometers (1000 Hz) that were securely placed on the sacrum, parallel to the lumbar spine, and strongly fixed with two Tarmak elastic straps (Decathlon Kipstadium, Tourcoing, France) (Figure 1a). The accelerometer’s position was unaltered and was checked among all running trials. A 5 min warm up at 3.3 m/s was performed before the trial, and the participants had a run-up to reach a constant speed. Each participant performed one trial of 120 m running, consecutively, with 40 m on each of the three different surfaces (synthetic track, grass, and concrete) at a 3.3 m/s running-speed, which was controlled with a GPS (Garmin Fore-Runner 630, Garmin Ltd., Olathe, KS, USA) by an operator who ran behind the participant (Figure 1b). Each participant used running shoes that they habitually used in their workouts and competitions to achieve their natural running technique. The surface order was randomized.

The data of accelerations in impacts were obtained using Qüiko software v. 0.905 (Real Track^®^, Almería, Spain), and filtered at 12 Hz using a Butterworth 4th order filter (by S. Butterworth, M.Sc., Admiralty Research Laboratory, Teddington, London, UK). This frequency was previously selected according to the residual analysis results [23].

The variables defined below were obtained during the middle four consecutive steps made during running on each surface:

- The total acceleration (TA), that is, the resultant acceleration obtained using the vectorial sum of the acceleration in each axis (ax, ay, az) registered during the impacts (measured in g).
- The mean total acceleration (MA), that is, the average of the total accelerations registered during the impacts (measured in g).
- The mean peak total acceleration (PA), that is, the average of the maximum total accelerations registered during the impacts (measured in g).
- The number of peaks of total acceleration in the impacts (NI), that is, the number of maximum total accelerations registered during the impacts. All the NI were grouped considering their magnitude of acceleration, within the following ranges: 0 to <1 g, 1 to <2 g, 2 to <3 g, 3 to <4 g, and 4 to <5 g.
- The number of running steps (NS), that is, the number of strides taken over 40 m.

### 2.3. Statistical Analysis

The selected variables were naturally log-transformed to satisfy the data normality assumptions (Kolmogorov–Smirnov test). A mixed linear model was used to compare the effect of the terrain (synthetic track, grass, and concrete) as a fixed effect and of the athletes as a random effect on the running acceleration in the impacts. Magnitude-based inferences [24] were determined from standardized differences (SD) between values to determine small, moderate, and large differences (values = 0.2, 0.6, and 1.2, respectively, [25]) with a 90% confidence interval (CI). All the calculations were carried out using the statistical software IBM SPSS Statistics for Windows, Version 24.0. (IBM Corp., Armonk, NY, USA), and Microsoft Excel spreadsheets (Microsoft Inc., Redmond, WA, USA); a confidence interval of 90% was used.

## 3. Results

Descriptive data of the MA, PA, NI, and NS of each surface for the synthetic track, grass, and concrete, respectively, are presented in Table 1. The mean total accelerations were higher on concrete than on the synthetic track and on grass, about 3.0–3.7%, respectively, and the mean peak total accelerations were about 3.6–5.6% higher, respectively. However, the number of peaks of total acceleration was lower on concrete than on the synthetic track and on grass, by about 5.5–8.9%, respectively; the number of steps taken during the trial was also 4.4–6.3% lower on concrete, respectively.

Table 1 shows that in each step, the runners reached around 2 peaks of total acceleration in the impacts (NI): 2.12 when running on the synthetic track, 2.15 on grass, and 2.09 on concrete.

Table 2 shows the average NI at different magnitudes of acceleration in the impacts on different surfaces. Although concrete scored lower than the synthetic track and grass for the 2 to <3 and 3 to <4 g number of peaks, it presented a higher value for the 4 to <5 g range. The NI of magnitude 2 to <3 g received for runners on concrete was 9.6% lower than the value for grass and 12.4% lower than that for the synthetic track; the value for grass was about 3% lower than that for the synthetic track. The NI of magnitude 3 to <4 g registered on concrete was 10.6% lower than the value on grass, and 5% lower than that on the synthetic track; the synthetic track value was 5.9% lower than that for grass. Lastly, the higher NI at 4 to <5 g on concrete was found to be around 36–37% higher than on the synthetic track and on grass.

**Figure 2:**
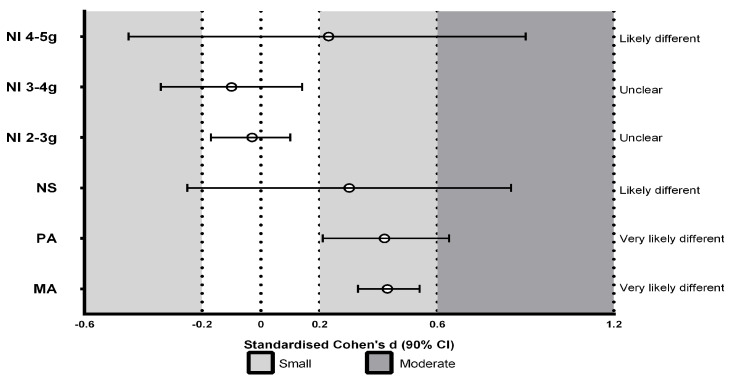
Standardized differences (SD) between synthetic track and concrete in the number of peaks of total accelerations in the impacts (NI) at different magnitudes of acceleration: 2 to <3 g; 3 to <4 g; 4 to <5 g, number of steps (NS), mean maximum (peak) accelerations (PA), and mean total acceleration (MA) in the impacts.

**Figure 3:**
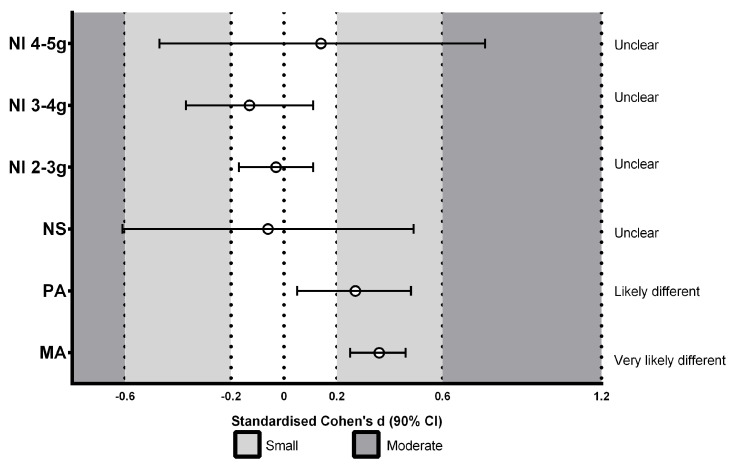
Standardized differences (SD) between grass and concrete in the number of peaks of total accelerations in the impacts (NI) at different magnitudes of acceleration: 2 to <3 g; 3 to <4 g; 4 to <5 g, number of steps (NS), mean maximum (peak) accelerations (PA), and mean total acceleration (MA) in the impacts.

**Figure 4:**
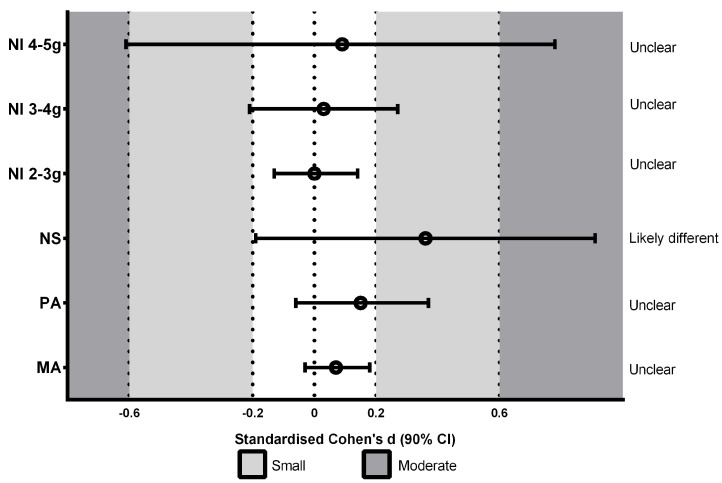
Standardized differences (SD) between synthetic track and grass in the number of peaks of total accelerations in the impacts (NI) at different magnitudes of acceleration: 2 to <3 g; 3 to <4 g; 4 to <5 g, number of steps (NS), mean maximum (peak) acceleration (PA), and mean total acceleration (MA) in the impacts.

Figure 2, Figure 3 and Figure 4 represent the effects of surface type on accelerometry-derived parameters: synthetic track compared to concrete (Figure 2), grass compared to concrete (Figure 3), and synthetic track compared to grass (Figure 4). The results reveal that concrete surfaces showed a greater PA and MA than other surfaces; small differences in PA were found from the synthetic track: SD 0.42 (0.21, 0.64) and grass: SD 0.27 (0.05, 0.48). Similarly, concrete presented small differences in MA from the synthetic track: SD 0.43 (0.33, 0.54) and grass: SD 0.36 (0.25, 0.46). Greater NS values were observed for the synthetic track; small differences from concrete (SD 0.30 (−0.25, 0.85)) were shown, as well as grass: SD 0.36 (−0.19, 0.91). Trivial differences were observed between concrete and grass. The NI values of 2 to <3 g and 3 to <4 g showed no significant differences between surfaces. The NI of 4 to <5 g of the magnitude of acceleration (which was greater for concrete) showed small differences between the synthetic track and concrete: SD 0.23 (−0.45, 0.9).

## 4. Discussion

In this study, a comparison of accelerations in impacts of amateur runners running at a constant velocity on three surfaces (concrete, synthetic track, and grass) was carried out. The results showed that the running impacts differed based on the type of surface, indicating that the runners possibly changed their running technique on the different surfaces.

Higher mean and peak total accelerations in the impacts were observed while the runner ran on concrete, as compared to the other two surfaces. The findings imply that the runner is subject to greater biomechanical loads on concrete, as shown in previous studies [5,6,26]. These differences could be due to the greater attenuation of impacts on synthetic tracks or grass.

The results indicate that upon each runner´s foot striking the ground, around two peaks of acceleration were generated with each step. Comparing the synthetic track and concrete (Figure 2), a higher number of peaks were obtained in the range of 4 to <5 g on concrete, and similar values were obtained for other ranges of accelerations. Although the effect of fatigue was not investigated in this study, previous research has shown that fatigue is known to cause a 50% increase in acceleration, so the leg is subject to higher impacts, and the overload injury risk increases [27]. This is important considering that popular long-distance races are run on concrete, in which the fatigue at the end of the race could produce higher acceleration impacts and thereby increase of the risk of injury.

Another important observation is that the number of steps was higher on the synthetic track and grass than on concrete (Table 1). This indicates that running technique changes depending on the terrain. Most runners are usually trained on synthetic tracks, and this has been considered the optimum surface for running. However, most popular races are run on concrete, and it appears that on this surface, runners adapt their technique, significantly reducing their stride frequency. Nevertheless, the results of this study show that this lower stride frequency provokes large-scale accelerations. This finding is in accordance with previous research, wherein it was shown that reducing the stride frequency increased the vertical acceleration of the tibia during early stance [28]. In distance running, an increase in vertical load and high tibial peaks of acceleration in the impacts are related to medial tibial stress syndrome [29].

A novelty of this study was the definition of the variable number of peaks of total accelerations in the impacts (NI), and its classification according to its magnitude. In addition, the location of the sensor could also affect the data registered. The majority of the studies placed accelerometers on the tibia; on the proximal anteromedial aspects of the right tibia [10]; on the anteromedial aspect of the distal third of the tibia [30]; on the lateral surface of the distal lower leg [17]; 8 cm above the medial malleolus on the right tibia [4]; on the tibial tuberosity of the dominant leg [13]; 8 cm above the medial malleolus on the distal anteromedial aspect of the tibia [19]; and on the distal half of the anteromedial aspect of the right tibia [14]. Some have placed accelerometers on the heel (above the midsole at the heel, [13]), on the head of the fifth metatarsal [14], the sacrum [16], trunk [5], and the forehead [10,17]. In the present study, the sensor was placed on the sacrum, closer to the center of mass (CM) wherein the effect of the impacts would be more attenuated than in the lower limbs; however, this positioning can also provide more global information on the effect of the impacts on the locomotor system of the runner. The sensor gives different results based on its position, and hence a consensus on the sensor location could help for comparing results from different surfaces.

Previous research in biomechanics has shown that running technique varies based on velocity in running [31] and gait [32]. The findings from this study indicate that there is a possibility that the type of surface also affects technique when running. Although this study gave interesting results, their interpretation must be carried out carefully. In this study, the velocity was controlled, and so were the distances run on each surface. Future studies could consider the effect of different velocities on different surfaces, and studying the same over longer distances could help us to analyze the effect of fatigue. The participants were asked to use the footwear that they normally use to achieve ecological validity, but the effect of footwear on the variability of data was not determined (no data on the level of use, time since purchase, or the quality of the footwear were taken). This is another limitation of this study that other studies from this line of research, or from podiatry, could remedy by identifying the time of use of the footwear, its degradation, its impact on different surfaces, the brand of the footwear, and whether or not it modifies running technique [33,34]. The large variability in the data at higher impact accelerations could be due to the different running techniques and foot strike patterns in amateur runners [30]. Future studies could also consider differences between men and women, as we know that biomechanical, hormonal and even social differences can be observed in this area [35,36]. In this research, due to the limited number of samples, we could not establish these groups to produce a solid comparison between men and women.

## 5. Conclusions

Given the increasing popularity of running, athletes must be trained to withstand different loads on different surfaces. Runners should train their technique on a combination of surfaces, and not just on a synthetic track. Running on a synthetic track and grass could be more suitable during training periods, as these surfaces produce fewer peak accelerations in impacts. Running on concrete could be limited to competitions in which this surface is usually chosen by the organizers of these events. On concrete, runners could try to increase their stride frequency to reduce the high acceleration in the impacts and, as a consequence, reduce the risk of injury. The hypothesis that running on concrete surfaces results in higher accelerations in impacts when the runner´s foot strikes the ground was confirmed; this indicates that prolonged training periods on such a surface could lead to an increased risk of injuries. Thus, runners are advised to use a combination of surfaces while preparing for competitions.

## Figures and Tables

**Table 1:** Mean and standard deviation for mean total accelerations (MA), mean peak total accelerations (PA), number of peaks of total acceleration in the impacts (NI), and number of steps (NS) in the trial.

	Synthetic Track	Grass	Concrete
Mean acceleration (g)	1.30 ± 0.1	1.31 ± 0.1	1.35 ± 0.1
Maximum acceleration (g)	3.68 ± 0.45	3.76 ± 0.48	3.90 ± 0.55
Number of peak accelerations	74.03 ± 12.51	76.83 ± 9.85	69.97 ± 10.39
Number of steps	34.90 ± 2.67	35.60 ± 3.94	33.37 ± 2.95

**Table 2:** Average number of peaks of total accelerations in the impacts (NI) grouped in three magnitudes of acceleration for the different surfaces.

Magnitude of Acceleration	Synthetic Track	Grass	Concrete
2 to <3 g	21.03 ± 18.26	20.4 ± 17.51	18.43 ± 16.73
3 to <4 g	17.57 ± 11.75	18.67 ± 11.21	16.70 ± 10.36
4 to <5 g	2.00 ± 5.62	2.03 ± 4.41	3.17 ± 5.73

## Data Availability

Not applicable.
